# Tantalum nitride films integrated with transparent conductive oxide substrates *via* atomic layer deposition for photoelectrochemical water splitting†
†Electronic supplementary information (ESI) available. See DOI: 10.1039/c6sc02116f



**DOI:** 10.1039/c6sc02116f

**Published:** 2016-07-05

**Authors:** Hamed Hajibabaei, Omid Zandi, Thomas W. Hamann

**Affiliations:** a Michigan State University , Department of Chemistry , 578 S Shaw Lane , East Lansing , Michigan 48824-1322 , USA . Email: hamann@chemistry.msu.edu

## Abstract

Tantalum nitride, Ta_3_N_5_, is one of the most promising materials for solar energy driven water oxidation. One significant challenge of this material is the high temperature and long duration of ammonolysis previously required to synthesize it, which has so far prevented the use of transparent conductive oxide (TCO) substrates to be used which would allow sub-bandgap light to be transmitted to a photocathode. Here, we overcome this challenge by utilizing atomic layer deposition (ALD) to directly deposit tantalum oxynitride thin films, which can be fully converted to Ta_3_N_5_ *via* ammonolysis at 750 °C for 30 minutes. This synthesis employs far more moderate conditions than previous reports of efficient Ta_3_N_5_ photoanodes. Further, we report the first ALD of Ta-doped TiO_2_ which we show is a viable TCO material that is stable under the relatively mild ammonolysis conditions employed. As a result, we report the first example of a Ta_3_N_5_ electrode deposited on a TCO substrate, and the photoelectrochemical behavior. These results open the door to achieve efficient overall water splitting using a Ta_3_N_5_ photoanode.

## Abstract 2

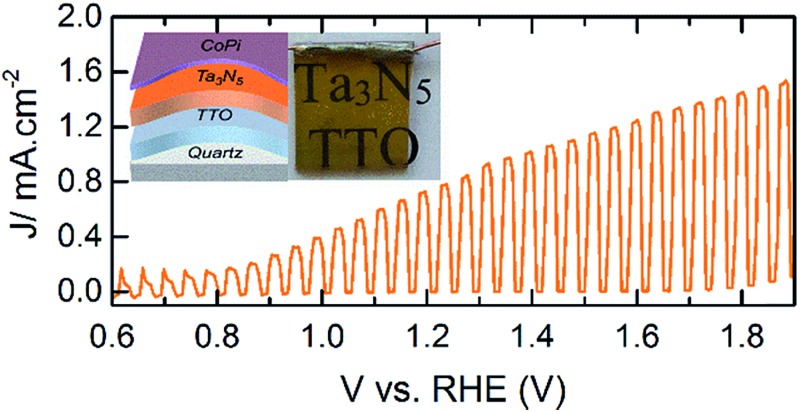
The first example of tantalum nitride electrodes on transparent conductive oxide substrates, which enables solar water splitting, is presented.

## Introduction

**Scheme 1:**
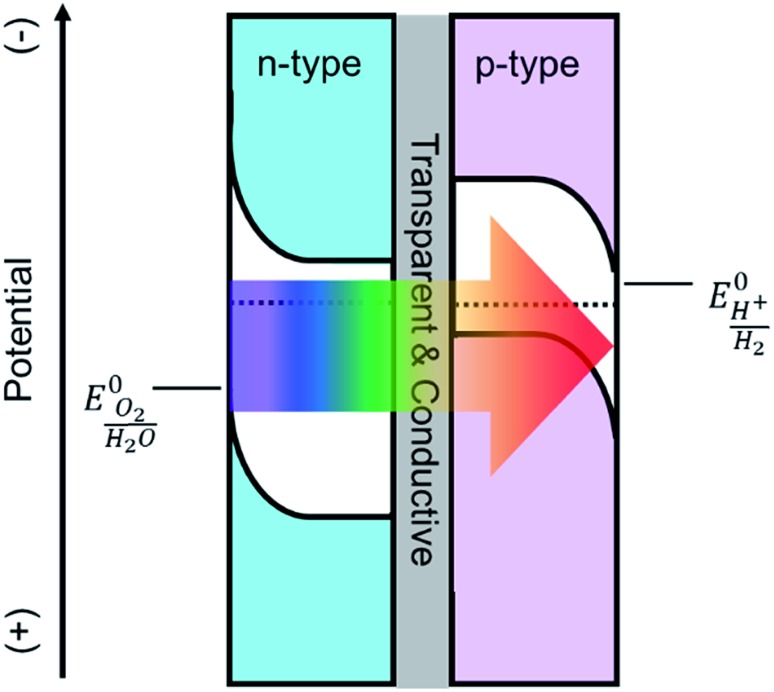
Depiction of a tandem cell configuration for overall water splitting composed of n-type, and p-type semiconductor connected in series with a transparent and conductive layer.

Solar driven photoelectrochemical (PEC) water splitting is a promising route to directly store solar energy in the chemical bonds of hydrogen. Due to the limitation of available materials capable of overall PEC water splitting, a tandem cell is likely required to efficiently convert solar energy into hydrogen.^1,2^ One promising tandem cell configuration is comprised of an n-type semiconductor as a photoanode to drive the oxygen evolution reaction that is electrically connected to a p-type photocathode to drive the hydrogen evolution reaction (Scheme 1). This type of PEC cell is advantageous as it allows researchers to independently investigate and optimize each half-cell. Although many semiconductor metal oxides have been proposed as a photoanode for solar water oxidation, the majority of their band gaps lie in the UV region which covers a negligible portion of solar spectrum.^3–6^ Metal oxide materials with narrower optical band gap and absorption edges that extend to the visible region, *e.g.* Fe_2_O_3_,^7,8^ WO_3_,^9,10^ and BiVO_4_,^11,12^ have therefore attracted a lot of attention. The state-of-the-art electrodes using these materials have produced promising water oxidation photocurrent densities, with the best examples producing approximately 5 mA cm^–2^ at 1.23 V *vs.* RHE. For example, Wang and co-workers recently employed a solution processed hematite photoanode in combination with an amorphous Si electrode to achieve overall water splitting at an efficiency of 0.91%. No metal oxide photoanode, however, has produced a photocurrent density that would enable achieving ∼10% water splitting efficiency.

Domen *et al*. have recently introduced a new class of nitride semiconductors, specifically tantalum nitride (Ta_3_N_5_), as promising alternative candidates to oxides for PEC water oxidation.^13–19^ In one impressive example, they demonstrated 1.5% efficient solar water splitting with Ba-doped Ta_3_N_5_ nanorods nitridized at 1000 °C for 2 h.^20^ Tantalum nitride is intrinsically an n-type semiconductor with an optical band gap of 2.1 eV that theoretically corresponds to a maximum photocurrent density of 12.5 mA cm^–2^.^21,22^ If it is coupled with an appropriate photocathode in a PEC tandem cell, it could perform unassisted water splitting at a solar-to-hydrogen efficiency of ∼15%.^23^ Strikingly, Li and coworkers recently reported a Ta_3_N_5_ photoanode on Ta foil prepared by ammonolysis at 950 °C for 6 h that produced a photocurrent density of ∼12.1 mA cm^–2^ at 1.23 V *vs.* RHE with a photocurrent onset potential of ∼0.7 V *vs.* RHE.^24^

Implementing a Ta_3_N_5_ photoanode in a tandem configuration to achieve efficient overall water splitting is hindered by the lack of a synthetic procedure to prepare Ta_3_N_5_ electrodes under conditions compatible with a transparent conductive oxide (TCO) substrate. Most of the studies on tantalum nitride (Ta_3_N_5_) share a similar synthetic route, beginning with the oxidation of Ta(0) to Ta(v), followed by ammonolysis at elevated temperatures (>800 °C) for long periods of time (>6 h), as noted in the best literature examples provided above. Despite the simplicity and great performance that comes with this method, there are multiple negative consequences. The initial oxidation of tantalum metal is very energy intensive. Importantly, the subsequent ammonolysis prevents the use of a TCO substrate as noted by others.^18^ In our lab, we found that when conventional TCOs such as FTO (F-doped SnO_2_), ITO (Sn-doped In_2_O_3_), and AZO (Al-doped ZnO) are exposed to ammonia at 600 °C or higher, they are reduced to metallic phases and become flaky with weak adhesion to the substrate. Consequently, their important properties of conductivity and transparency are lost. Therefore, Ta_3_N_5_ is commonly prepared on Ta foil which excludes the applicability of the Ta_3_N_5_ as a photoanode in a tandem configuration (Scheme 1), since the substrate is not transparent to subbandgap light. Furthermore, high temperature ammonolysis makes it difficult to control the morphology, interfaces and the inherent properties of this semiconductor.

In order to overcome these issues, we synthesized Ta-doped TiO_2_ (TTO) films *via* atomic layer deposition (ALD) which we found to be a stable TCO in reducing atmospheres. In addition, to circumvent the high temperature ammonolysis, ALD was also used to directly deposit thin films of Ta_3_N_5_ on the TTO substrates. While initial as-deposited films are primarily amorphous TaO_*x*_N_*y*_, these films can be nitridized to Ta_3_N_5_ at far more moderate nitridation conditions, *i.e.* 750 °C for 30 minutes, compared to previous reports where hours (>6 h) of nitridation at temperatures higher than 800 °C were necessary. The photoelectrochemical properties of the Ta_3_N_5_ films deposited on TTO were investigated and the PEC water oxidation performance was analyzed. The excellent material control reported here allowed for a detailed material structure–function relationship to be determined and a path to improved performance elucidated.

## Experimental

### Film preparation

Thin films of Ta-doped TiO_2_ TCO films were prepared on quartz substrates (Advalue Technology) by alternating the deposition of TiO_2_ and TaO_*x*_ with four different ratios of TaO_*x*_ : TiO_*x*_ ALD cycles, 1 : 200, 1 : 150, 1 : 100, and 1 : 50, to modify the dopant concentration. TiO_2_ was deposited using a modified literature procedure;^25^ briefly, titanium isopropoxide (99.9%, Aldrich) was heated to 80 °C and pulsed for 2 s. After purging for 10 s, water was pulsed for 15 ms followed by purging for another 10 s. The growth rate of TiO_2_ at 250 °C was found to be 0.2 Å per cycle. The deposition of the TaO_*x*_ sub-cycles is described below. The as-deposited TTO films were subsequently annealed under an ammonia atmosphere at 750 °C for 30 minutes with a heating rate of 35 °C min^–1^ and cooled down to the room temperature by opening up the top cover of the tube furnace.

TaO_*x*_N_*y*_ and TaO_*x*_ films were deposited on quartz, silicon (University Wafer, with ∼16 Å native SiO_2_) or the TTO coated quartz substrates described above using ALD (Savannah 200, Cambridge Nanotech Inc). All substrates were sequentially sonicated for 15 minutes in soap, DI water and isopropyl alcohol, then blown dry under a nitrogen flow and loaded into the ALD chamber. High purity nitrogen was used as a carrier gas, which was further dried and deoxygenated by in-line molecular sieves 3 Å (Sigma Aldrich) and an O_2_ scrubber (Restek), respectively. Throughout the deposition, the N_2_ flow rate was adjusted at 5 SCCM, providing a constant pressure of ∼350 mTorr. Pentakis(dimethylamine)tantalum(v), Ta(N(CH_3_)_2_)_5_ (PDMAT), (99.9%, Aldrich) was used as the tantalum precursor. Monomethyl hydrazine, CH_3_NHNH_2_ (MMH), (99.9%, Aldrich) or DI water (Millipore, 18 MΩ m) were used as the co-reactants. The tantalum precursor, PDMAT, was kept at 90 °C and consecutively pulsed 5 times for 2 s duration with 10 s purging in between pulses. The MMH and DI water co-reactants were kept at ambient temperature. Nitridation or oxidation was performed by a 15 ms pulse of MMH or water followed by purging for 15 s to complete one ALD cycle. Films were annealed in an ammonia atmosphere at 750 °C for 30 min to complete the nitridation and crystallize the films.

### Film characterization

Film thicknesses were determined *via* spectroscopic ellipsometry (SE) using a Horiba Jobin Yvon, Smart-SE instrument. X-ray photoelectron spectroscopy (XPS) was performed with a Perkin Elmer Phi 5600 ESCA system using a monochromatic Mg Kα source to illuminate the sample at a takeoff angle of 45°. Survey scans of 0–1100 eV binding energy and detailed scans for C 1s, O 1s, N 1s and Ta 4f, Ti 2p regions were measured for all samples. The binding energies were corrected in reference to C 1s peak (284.8 eV) and Shirley background subtraction was performed for fitting for each sample. Absorbance spectra were collected on a Perkin Elmer Lambda35 UV-vis spectrometer equipped with a Labsphere integrating sphere. Raman spectra were recorded using a LabRam Armis, Horiba Jobin Yvon instrument equipped with a 532 nm laser and a ×50 microscope to focus the laser on the film surface. X-ray diffraction (XRD) patterns were obtained on a Bruker D8 Advanced diffractometer using Cu radiation with a Kα1 wavelength of 1.5418 Å. 4-Probe electrical measurements were performed using a computer controlled Pro4-440N system equipped with Keithley 2400, and Pro4 software. The film thickness was also measured by cross-section SEM (Carl Zeiss Auriga, Dual Column FIBSEM) and was taken at a tilt angle of 90°.

All electrodes were coated with the Co-Pi co-catalyst *via* photoelectrodeposition prior to carrying out further PEC measurements. The Co-Pi co-catalyst was deposited in a solution with 0.5 mM Co(NO_3_)_2_ in a 0.1 M potassium phosphate buffer at pH 7 at a constant potential of 1.06 *vs.* RHE for 180 s under AM 1.5 G simulated sunlight. A Ag/AgCl and high surface area platinum mesh were used as the reference and counter electrodes, respectively.

Photoelectrochemical measurements were made with an Eco Chemie Autolab potentiostat coupled with Nova electrochemical software. The light source was a 450 W Xe arc lamp (Horiba Jobin Yvon). An AM 1.5 solar filter was used to simulate sun light at 100 mW cm^–2^ (1 sun). All the photoelectrochemical measurements were performed by shining light on the electrodes through electrolyte. Current–voltage curves were measured using a scan rate of 10 mV s^–1^. The incident light was chopped using a computer controlled Thor Labs solenoid shutter. Electrodes were masked with a 60 μm Surlyn film (solaronix) with a 0.28 cm^2^ hole which was adhered to the electrode by heating to 120 °C. The protected electrodes were clamped to a custom made glass electrochemical cell with a quartz window. A homemade saturated Ag/AgCl electrode was used as the reference electrode and was frequently calibrated to a commercial saturated calomel electrode (Koslow Scientific). Potentials *vs.* Ag/AgCl were converted to reversible hydrogen electrode (RHE) by the equation *E*_RHE_ = *E*_Ag/AgCl_ + 0.197 V + (0.059 V)pH. An aqueous solution of 0.5 M K_2_HPO_4_ was used as the electrolyte. The pH of the electrolyte was adjusted to 13 by adding KOH. A high surface area platinum mesh was used as the counter electrode.

## Results

### ALD of TTO

ALD was used to deposit Ta-doped TiO_2_ (TTO) on quartz substrates. Different Ta concentrations were introduced by varying the relative number of ALD cycles of TiO_2_ and TaO_*x*_. Samples with TaO_*x*_ : TiO_2_ ALD sub-cycle ratios of 1 : 50, 1 : 100, 1 : 150 and 1 : 200 were prepared to produce a series of decreasing Ta dopant concentrations in TiO_2_. In addition, pure TiO_2_ films were prepared as control substrates. The total number of cycles were controlled to keep the final film thickness constant at 100 nm. Energy dispersive spectroscopy (EDS) was used to determine the resultant concentration of Ta in TiO_2_. Since the Si (Kα: 1.739 eV) signal from the quartz coincides with Ta (M: 1.809 keV), TTO films were also deposited on Al (Kα: 1.486 eV) substrates which have a well-separated EDS peak (Kα(Al): 1.486 eV).^26^ The EDS spectra of these films with different concentration of Ta are shown in Fig. S1.† The Ta concentration in the 1 : 200 film was below the detection limit of the instrument, so it was not included in this plot, however, all the observed signals for the other three films are readily assigned to Ta, Ti, O, and Al (substrate). As shown in Fig. S1,† the atomic percentage of Ta was found to increase linearly with the ALD sub-cycle ratio of TaO_*x*_ : TiO_2_. The atomic percentages of Ta in the films, then were calculated from a linear fit of these data and used to assign the following percentages of Ta contained in the films: 5.0 (±0.32), 2.5 (±0.16), 1.67 (±0.11) and 1.25 (±0.08). We note that the actual concentration of Ta contained in the TTO films does not correspond simply to the pulse ratios of Ta and Ti precursors. The difference can largely be accounted for by the different growth rates: ∼0.25 Å per cycle for TiO_2_ compared to ∼0.79 Å per cycles for TaO_*x*_, *vide infra*.

The resistivity of the as-deposited Ta-doped TiO_2_ films on quartz were on the order of MΩ cm. In addition, consistent with a previous study, we observed that when the Ta-doped TiO_2_ films were annealed in air or oxygen, they became more insulating.^27^ Prior examples of Ta-doped TiO_2_ were prepared at low oxygen pressure, *e.g.* 10^–5^ Torr, or the films were annealed in vacuum.^27–29^ Since our ultimate goal is to realize TCO films coated with Ta_3_N_5_, which may have to be annealed under ammonia, *vide infra*, all TCO films were annealed under a reducing ammonia atmosphere at 750 °C for 30 minutes.

**Fig. 1:**
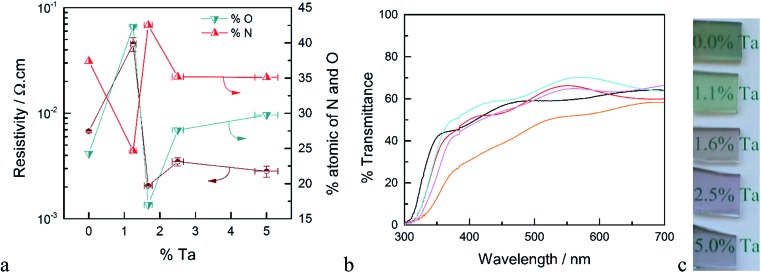
(a) Resistivity (brown) and atomic% of O (blue) and N (red) of Ta-doped TiO_2_ as a function of % Ta. (b) Optical transmittance of Ta-doped TiO_2_ films, un-doped TiO_2_ (orange), 5.0% Ta (black), 2.5% Ta (red), 1.67% Ta (cyan) and 1.25% Ta (pink). (c) Photographs of the TTO films after annealing in ammonia at 750 °C for 30 min.

XPS measurements were performed on samples deposited on quartz both before and after annealing in ammonia. The surface concentration of Ta for the as-deposited films is higher compared to the results from EDS measurements (see Fig. S2†). Since XPS is a surface sensitive technique, this higher apparent concentration of Ta may be attributed to the fact that the deposition of TaO_*x*_ was the last ALD cycle of these films. After annealing in ammonia, however, the atomic ratio of Ta/Ti determined by XPS was within error of the ratio determined by EDS on the as-deposited samples. Thus, annealing allows Ta to diffuse and be homogeneously distributed throughout the film. We therefore take the surface compositional analysis done by XPS after annealing as a good approximation of bulk composition. Details of the XPS analysis of as-deposited and annealed TTO films with different concentrations of Ta are discussed following Fig. S3 in the ESI.† The atomic percentages of oxygen and nitrogen as a function of Ta concentration after annealing in ammonia are shown in Fig. 1a. After annealing in ammonia the atomic percentage of O decreased and a new N signal emerged which indicates oxygen is substituted by nitrogen in the films. Thus, the annealing step results in TiO_2_ co-doped with Ta and N. Interestingly, at high concentration of Ta, *i.e.* ∼5%, another N signal is detectable which can be assigned to a Ta–N bond. Further, the Ta signal from the same film shows two types of Ta present in the films. Therefore, we attribute this to the formation of TaN_*x*_ as a separate phase at high Ta concentrations. This observation is supported by the XRD results of the films and the resistivity of the films discussed below.

The XRD diffraction patterns of all annealed samples were unambiguously assigned to anatase TiO_2_. A detailed analysis of the XRD patterns of the N- and Ta- co-doped TiO_2_ films with different Ta concentrations is discussed in the ESI following Fig. S4.† Depending on the dopant concentration, however, the peak positions of anatase are shifted to the lower angles which indicates an increase in cell volume as expected from doping Ta into TiO_2_.^30^

The resistivity of the TTO films as a function of the concentration of Ta is shown in Fig. 1a. The resistivity decreases sharply with introduction of Ta, reaching a minimum for the film with 1.6% Ta. This is ∼3 times smaller than the optimum Ta concentration reported in the literature.^28,31^ The main difference between the Ta-doped TiO_2_ synthesized in this study to those reported in literature is the annealing atmosphere. As noted above, the use of ammonia as the reducing atmosphere results in TiO_2_ films co-doped with Ta and N. As depicted in Fig. 1a, the resistivity of the films has a strong correlation to the atomic concentration of oxygen and nitrogen. The film without Ta exhibits a surprisingly low resistivity which results from the formation of nitrogen-doped TiO_2_ or segregation of metallic TiN phases. The lowest resistivity occurs for the film with 1.6% Ta, which has the highest concentration of nitrogen and the lowest concentration of oxygen, *i.e.* the highest concentration of oxygen vacancies. Based on the formal charge of oxygen and nitrogen, it can be inferred that substitution of oxygen with nitrogen induces an increase in the concentration of oxygen vacancies. On the other hand, substitution of Ti^4+^ with Ta^5+^ may reduce the number of oxygen vacancies. Therefore, co-doping of N and Ta into TiO_2_ may have an opposing influence on carrier concentration and conductivity, which explains the difference between the optimal doping concentration found here compared to prior reports.^27^

The optical transmittance of un-doped and Ta-doped TiO_2_ thin films after annealing in ammonia is shown in Fig. 1b. Note that these transmittance values were not corrected for reflectance, which accounts for ∼25% loss of incident photons (Fig. S5†). The transmittance of the TiO_2_ without Ta was below 50% in the visible region, which is in line with numerous reports of N-doped TiO_2_.^32,33^ The substitution of oxygen with nitrogen introduces new states in the band gap which results in absorption edge tailing to the visible region. Upon Ta-doping, however, the average transmittance values in the visible region are increased with a maximum transmittance value of ∼70% for 1.6% Ta doped in TiO_2_.

### ALD of Ta_3_N_5_

**Fig. 2:**
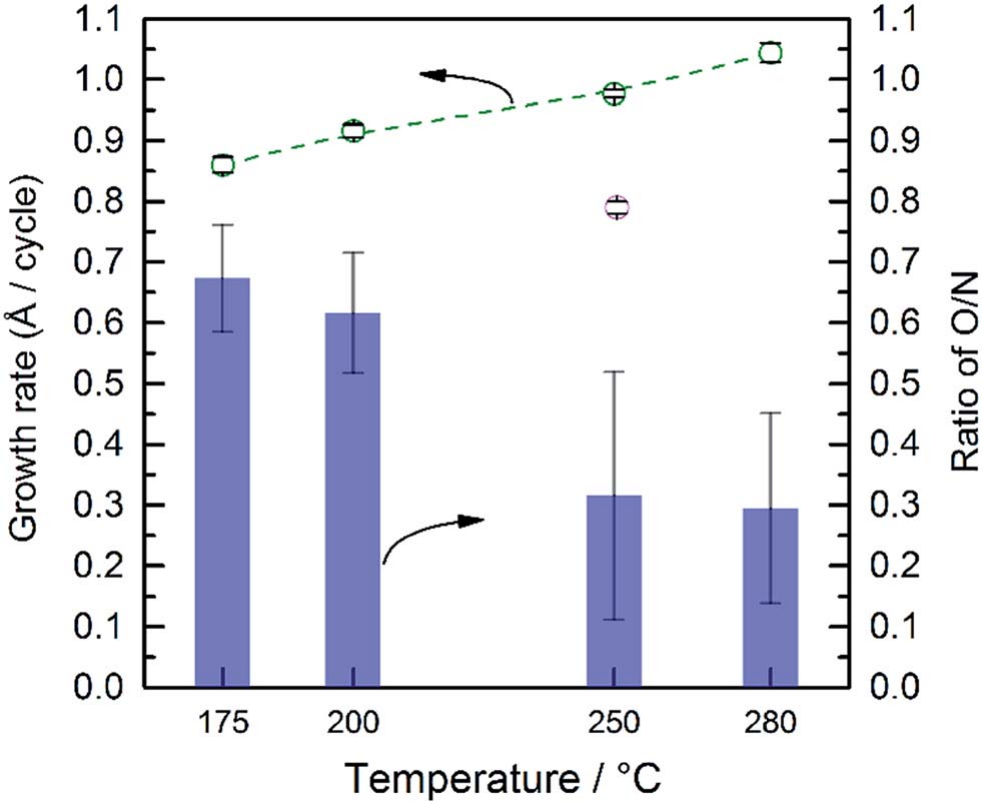
Growth rate of TaO_*x*_N_*y*_ (green) and TaO_*x*_ (pink) as a function of deposition temperature. Also shown are the ratios of atomic concentrations of O/N (purple) as a function of the deposition temperature found from the EDS analysis.

TaO_*x*_ films were deposited from 50–500 ALD cycles at 250 °C. The resultant film thickness increases linearly with the number of ALD cycles (Fig. S6†). The growth rate was found to be 0.79 Å per cycle, which is in good agreement with the previous report of the ALD deposition of TaO_*x*_ (0.85 Å per cycle).^34^ The ALD of tantalum nitride using PDMAT and MMH has previously been studied, where an ALD deposition temperature window between 200 and 300 °C was found with a growth rate of ∼0.3 Å per cycle.^35^ It has been previously reported that PDMAT suffers from thermal decomposition at temperatures above 300 °C, therefore, to avoid the decomposition of the precursor and ensure an ALD process, 280 °C was used as the maximum deposition temperature.^34,35^ Interestingly, while we confirmed an ALD temperature window over 175 to 280 °C, we found a growth rate approximately three times larger with a small temperature dependence. Plots of thickness *vs.* number of ALD cycles are provided in Fig. S7.† From the slope of these plots, the growth rates were found to reproducibly vary from 0.86 Å per cycle at 175 °C to 1.04 Å per cycle at 280 °C, as shown in Fig. 2.^35,36^ These growth rates were confirmed by cross section SEM measurements of the films grown with 1000 ALD cycles at 175 and 280 °C, which are shown in Fig. S8.† The images indicate a ∼28 nm difference in film thicknesses which is consistent with the different growth rates displayed in Fig. 2.

In addition to the growth rate, the temperature affected the composition of the deposited films. The bulk composition of the as-deposited films was analyzed by EDS (Fig. S9†). Silicon with ∼16 Å SiO_2_ was used as the substrate. The Ta and Si signals overlap which prevents accurate determinations of these individual elements. The atomic percentages of nitrogen and oxygen were calculated based on the signal of these two elements and are shown in Fig. 2. Oxygen was detected in all films. We note that only a minimal amount of O can be attributed to the ∼16 Å SiO_2_ substrate since the film thicknesses are ∼100 nm. Thus, despite the lack of oxygen in either ALD precursors, and the use of high purity nitrogen as a carrier gas, all deposited films are actually amorphous, TaO_*x*_N_*y*_. Thus, there must be some source of oxygen which we were not able to fully eliminate despite significant efforts to control the ALD atmosphere. Further, as the deposition temperature increases from 200 to 250 °C there is a change in the relative percentage of oxygen and nitrogen; the relative amount of O compared to N decreases from ∼65% to ∼25%.

**Fig. 3:**
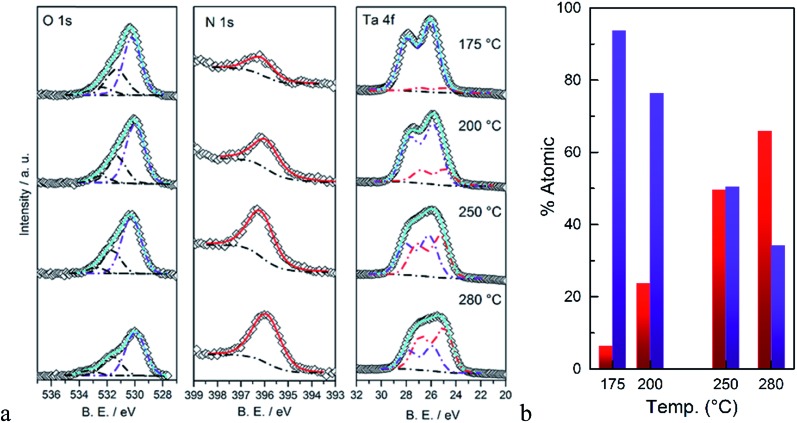
(a) XPS signals of O 1s, N 1s, and Ta 4f, (b) calculated atomic percentages of Ta–N (red) and Ta–O (blue) as a function of the deposition temperature.

The surface composition of the as-deposited films was also analyzed by XPS. Fitted spectra are shown in Fig. 3. As the deposition temperature increases, the N 1s signal grows and it can only be fitted to a single Ta–N peak. The oxygen signal was fitted to three peaks. Two peaks with binding energies >531 eV were assigned to carbon species, *i.e.* C–OH and C=O groups. The peak at 529–530.5 eV was assigned to the Ta–O group which was correlated to the Ta 4f peak. The peak positions of Ta 4f_7/2_ and Ta 4f_5/2_ strongly depend on the immediate surrounding atoms, *e.g.* ∼26.6 and 28.5 eV for Ta–O and 25.0 eV and 26.9 eV for Ta–N, respectively.^37^ Therefore to avoid complexity arising from carbon species, the surface atomic percentages of Ta–O and Ta–N were estimated from the Ta 4f peaks (Fig. 3b). As it can be seen, at lower temperatures the film is mostly composed of Ta–O groups. On the other hand, at higher deposition temperatures, the Ta–N becomes the dominant composition. This result is in line with the EDS analysis discussed earlier. These combined results are also in agreement with the previous study by Ritala *et al.* who studied the deposition of thin films of Ta_3_N_5_ at temperatures from 200 to 500 °C *via* ALD using TaCl_5_ and NH_3_ as the reactants.^38^ Their results showed that the composition of the films was strongly correlated to the deposition temperature and the concentration of oxygen was decreased from 25 to ∼5% as the deposition temperature increased from 200 to 500 °C.

The composition and growth rates of the films deposited at 280 and 250 °C are similar; since we found 280 °C to be the edge of the ALD temperature window, all the subsequent depositions of TaO_*x*_N_*y*_ were performed at 250 °C unless otherwise mentioned. A lack of diffraction peaks in XRD and phonon modes in the Raman spectrum of as deposited films indicate that these films are in fact amorphous TaO_*x*_N_*y*_ (Fig. S10†). Therefore, to improve the crystallinity and to modify the composition, they were annealed ammonia. There are three parameters which control the results of annealing; temperature, time and flow rate of ammonia. It was found that the optimum conditions (details discussed surrounding Fig. S11–S13†) to form pure crystalline Ta_3_N_5_ films from the as-deposited films is ammonolysis at 750 °C for 30 min with an ammonia flow rate of ≥200 mL min^–1^. It is worth noting that both ALD deposited thin films of TaO_*x*_ and TaO_*x*_N_*y*_ were nitridized to Ta_3_N_5_ (Fig. S14†) at far more moderate conditions compared to previous reports.^20,39,40^

Four TaO_*x*_N_*y*_ films of different thicknesses were deposited on quartz followed by ammonolysis at 750 °C for 2 hours. Based on the XRD patterns of the films (Fig. S15†), they can all be unambiguously matched to Ta_3_N_5_. The thicknesses of Ta_3_N_5_ films were evaluated *via* both cross section SEM and SE (Fig. S16†). As shown in Fig. S16d,† the growth rate found by both methods are in good agreement. However, the growth rate of pure Ta_3_N_5_, *i.e.* ALD deposition followed by ammonolysis, was ∼0.77 Å per cycle while the growth rate of the as-deposited films is ∼1.0 Å per cycle. This discrepancy in the growth rates is due to the fact that the as-deposited films are amorphous TaO_*x*_N_*y*_, whereas ammonolysis transforms the films to crystalline Ta_3_N_5_ which has 22% smaller molar volume per Ta atom than Ta_2_O_5_.^41,42^

**Fig. 4:**
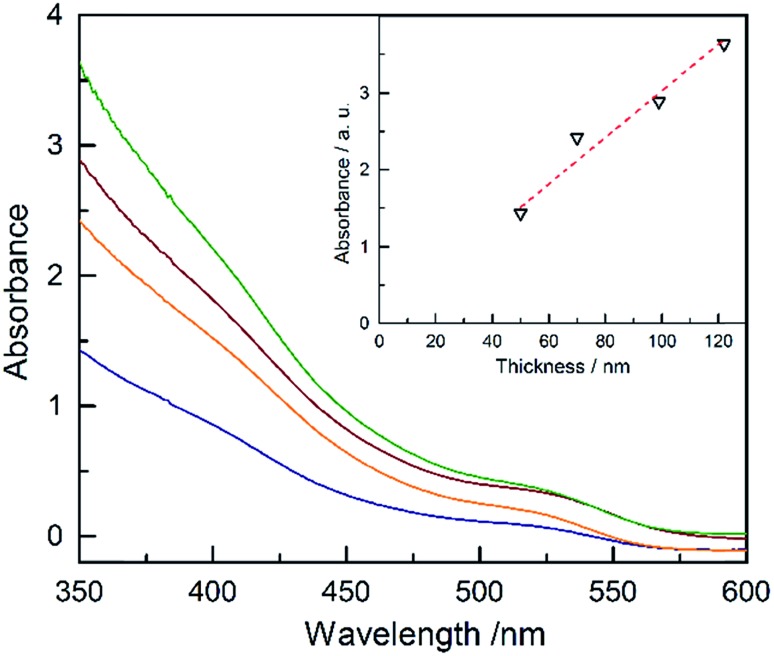
Plots of the absorbance of the thin films of Ta_3_N_5_ on quartz with different thicknesses: 50 nm (blue), 70 nm (orange), 99 nm (red), and 122 nm (green). The inset is the absorbance at 350 nm *vs.* the thickness of the films.

The absorbance of Ta_3_N_5_ as a function of the thickness is plotted in Fig. 4 (absorptance, transmittance and reflectance are shown in Fig. S17†). The absorbance was corrected for the substrate using a previously developed model.^43^ The absorbance scales linearly with the film thickness confirming a linear growth of tantalum nitride by ALD/ammonolysis. The absorption coefficient, *α*(*λ*) (cm^–1^), was calculated from absorbance using the average film thicknesses from SEM and SE (Fig. S18a†). Ta_3_N_5_ has two optical transitions, located at ∼2.10 eV and ∼2.50 eV. A recent study on optoelectronic properties of Ta_3_N_5_ suggests that both electronic transitions of Ta_3_N_5_ are direct.^44^ The corresponding Tauc plot for direct transitions is shown in Fig. S18b.†

**Fig. 5:**
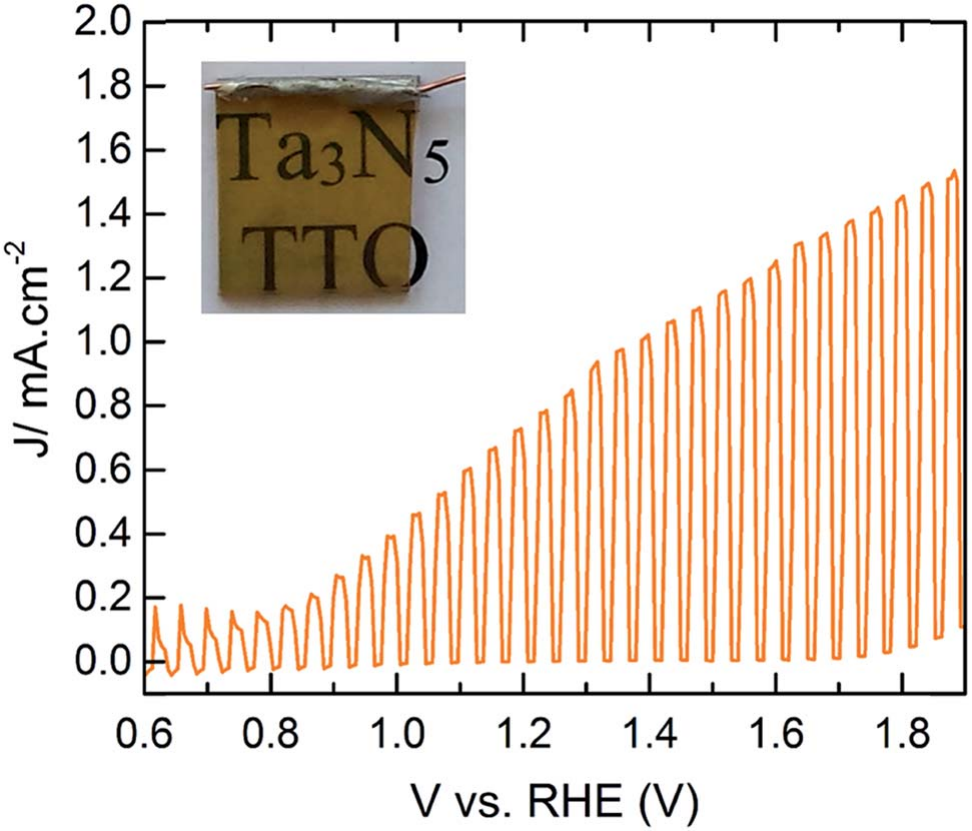
PEC performance of CoPi modified Ta_3_N_5_ (∼75 nm) on TTO with 1.6% Ta concentration under 1 sun illumination. The inset is the photograph of the working electrode.

To study the PEC performance, ∼75 nm (1000 cycles) of TaO_*x*_N_*y*_ was deposited on 100 nm TTO films with different Ta concentrations, followed by ammonolysis at 750 °C for 30 minutes. Attempts to increase the ammonolysis time to 2 hours resulted in transformation of the anatase-TiO_2_ to rutile-TiO_2_ in the TTO, based on the XRD patterns of the films, which resulted in an electrode with negligible photocurrent. Our initial results produce a photocurrent density of ∼0.77 mA cm^–2^ at 1.23 V *vs.* RHE with an onset photocurrent potential of ∼0.8 V *vs.* RHE. The PEC performance of these electrodes is strongly correlated to the conductivity of the TTO substrates. The photocurrent response of the electrodes at 1.23 V *vs.* RHE as a function of Ta concentration is shown in Fig. S21.† Remarkably, the observed photocurrents are in total agreement with the conductivity of TTO shown in Fig. 1a. This performance falls short of the recent report by Li and coworkers,^24^ who reported a photocurrent density of ∼12.1 mA cm^–2^ at 1.23 V *vs.* RHE with a photocurrent onset potential of ∼0.7 V *vs.* RHE for the electrode prepared on a Ta foil which was nitridized under ammonia at 950 °C for 6 h. Van de Krol *et al.* also recently studied the formation of Ta_3_N_5_ as a function of ammonolysis conditions on Pt foil.^14^ The maximum photocurrent density of ∼1.1 mA cm^–2^ at 1.23 V *vs.* RHE with an onset photocurrent potential of ∼0.9 V *vs.* RHE was found for the Ta_3_N_5_ film prepared at 800 °C for 10 h with the addition of IrO_2_ cocatalysts. To the best of our knowledge, however, this is the first report of PEC water oxidation of Ta_3_N_5_ on any TCO (Fig. 5).

Finally, since we have not yet eliminated the ammonolysis step in the synthesis, we compared the behavior of the TaO_*x*_N_*y*_ deposited films to TaO_*x*_. 40 nm of TaO_*x*_ was deposited on the best TTO (1.6% Ta) followed by ammonolysis at 750 °C for 30 minutes. The transmittance/reflectance spectra of the corresponding films are compared to that of the TaO_*x*_N_*y*_-derived film in Fig. S22.† The TaO_*x*_-derived film is colorless with a take-off transmittance at ∼450 nm. On the other hand, the TaO_*x*_N_*y*_-derived film is orange with a take-off transmittance at ∼590 nm which corresponds to the known band gap of Ta_3_N_5_, *i.e.* 2.1 eV, discussed above. The PEC performance of these electrodes are compared in Fig. S23.† The TaO_*x*_-derived film shows negligible photocurrent superimposed on a large dark current. Therefore, it can be concluded that the TaO_*x*_-derived films require harsher nitridization conditions (higher temperature and longer durations) where the TCO is not chemically stable.

## Conclusions

The realization of photoactive Ta_3_N_5_ films on a TCO electrode was demonstrated for the first time. This required two breakthroughs. First, we established the ALD of TTO and found that it is structurally and chemically stable (unlike conventional TCO materials including FTO, ITO and AZO) under the reducing atmosphere employed (ammonolysis at 750 °C for 30 min), and can therefore be used as a conductive transparent layer for tantalum nitride electrodes. The TTO films are not able to withstand harsher nitridation conditions required for the conversion of TaO_*x*_ to Ta_3_N_5_, however, which is the synthetic route of all prior examples of Ta_3_N_5_ photoelectrodes. Therefore, the second necessary breakthrough consisted of the direct deposition of TaO_*x*_N_*y*_ films *via* ALD which can be crystallized and completely converted to Ta_3_N_5_ under sufficiently milder ammonolysis conditions to maintain the TCO properties. The resultant Ta_3_N_5_ films on TTO showed promising solar water oxidation performance, especially considering that the films are quite thin and not yet optimized. We found that the performance of the photoelectrodes correlated to the conductivity of the TCO. Thus, it would be beneficial to utilize state-of-the-art TCOs such as FTO. Since these are not stable under even the mildest ammonolysis procedures utilized here, it would clearly be advantageous to directly deposit crystalline Ta_3_N_5_ films under sufficiently mild conditions on a TCO, which do not require a subsequent annealing (thus ammonolysis) step. While we are actively working on such an ideal synthetic method, the results reported herein represent a significant step towards realizing a high-efficiency solar water oxidation electrode which can be employed in a tandem configuration.

## Author contributions

The experiments were designed by H. H., O. Z. and T. W. H. The experiments were carried out by H. H. and O. Z. The manuscript was prepared by H. H. and T. W. H. All authors have approved the final version of the manuscript.
