# Supplementary material for: Tantalum nitride films integrated with transparent conductive oxide substrates via atomic layer deposition for photoelectrochemical water splitting
Source: Chem Sci. 2016 Jul 5;7(11):6760–7. doi: 10.1039/c6sc02116f (PMC5363780; doi:10.1039/c6sc02116f)
Supplement: Supplementary file 1 [file SC-007-C6SC02116F-s001.pdf]

Electronic Supplementary Material (ESI) for Chemical Science.
This journal is © The Royal Society of Chemistry 2016

SUPPORTING INFORMATION

Tantalum Nitride Films Integrated with Transparent Conductive Oxide Substrates via Atomic Layer Deposition for Photoelectrochemical Water Splitting

Hamed Hajibabaei, Omid Zandi, Thomas W. Hamann*

Michigan State University, Department of Chemistry

578 S Shaw Lane East Lansing, Michigan 48824-1322, United States

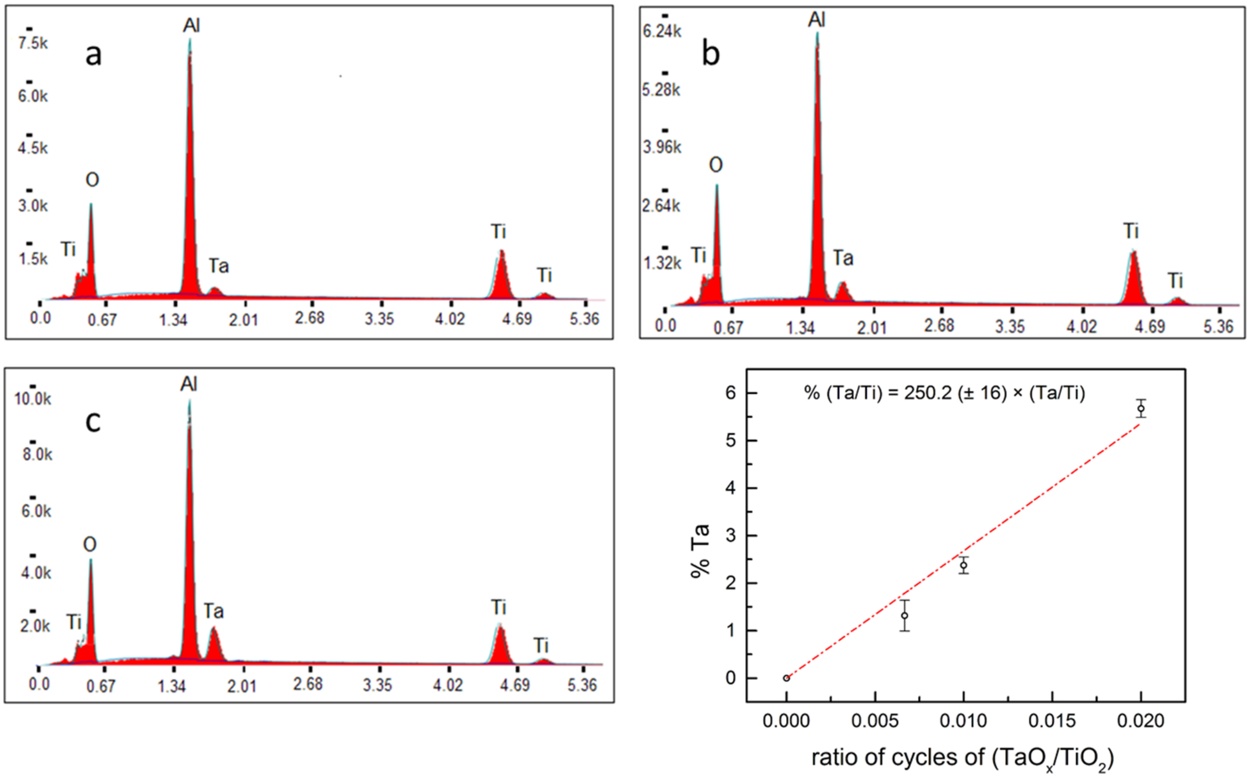

Figure S1. EDS spectrum of Ta-doped TiO_2 as a function of ratio of cycles of TaO_x to TiO_2, a) 1:150, b) 1:100, and c) 1:50. d) Atomic percentage of Ta as a function of ratio of cycles of TaO_x:TiO_2.

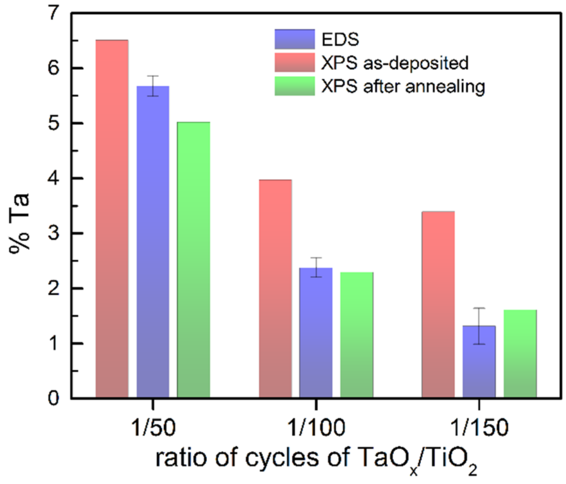

Figure S2. Comparison of atomic percentage of Ta as a function of ratio of cycles of TaO_x/TiO_2 found by EDS and XPS.

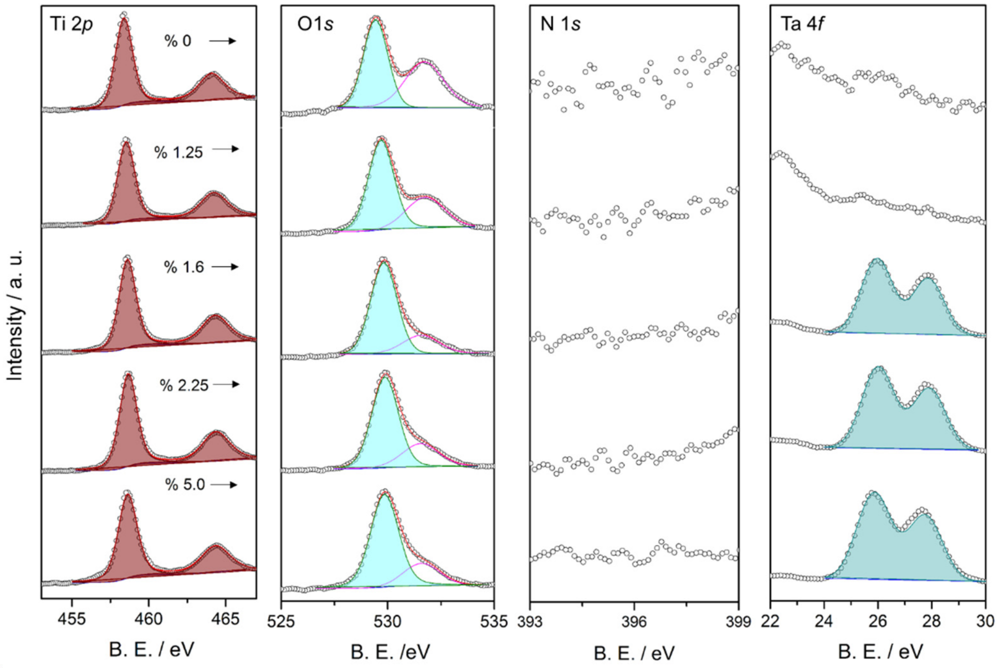

a

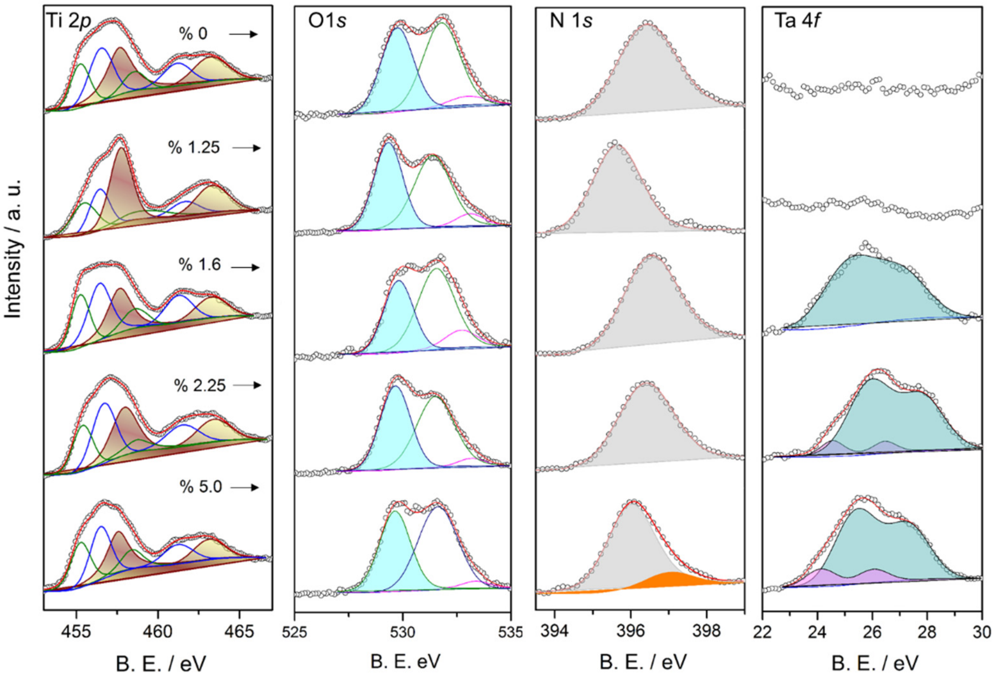

b

Figure S3. XPS spectra of Ta-doped TiO_2 as a function of % Ta doping, a) as deposited, b) after annealing in ammonia at 750 °C for 30 min.

The as-deposited films are only comprised of Ti, O and, Ta. After annealing in ammonia, nitrogen signal emerged. The main features of XPS of Ta-doped TiO_2 would be better captured by analyzing each individual elements before and after annealing in ammonia.

Ti: The Ti main peak before and after annealing in ammonia were located at the same position. Upon annealing in ammonia, however, two new peaks associated to Ti-O-N (blue) and Ti-N (green peak) groups were appeared which confirms that beside Ta, N is also doped into TiO.[3–5]

O: As expected, oxygen peak (metal oxygen bond) at 29-30 eV was not affected by annealing in ammonia, however, the atomic percentage of oxygen was changed by both annealing in ammonia and the atomic percentage of Ta. The other Oxygen peaks were assigned to C=O and C-OH groups.

N: As it can be seen, the as-deposited films did not have any nitrogen, however, after annealing in ammonia the nitrogen signal was emerged. At high concentration of Ta, there is a small shoulder (orange peak), therefore, it was considered as a different type metal-N bonding.

Ta: Before annealing in ammonia, there was only one type of Ta present which can be associated to Ta-O groups. Upon annealing in ammonia, however, Ta signals became broadened which is an indication of doping of Ta into the structure with a range of atomic interaction with its neighbors. The Ta signal for the film with high concentration of Ta, i.e. %5 Ta, could not be fitted to one peak, and it was fitted to two peaks. Therefore, there are two types of Ta present. This observation is in line with the extra N peak for the film with high concentration of Ta. Then it was hypothesized that at high % Ta doping, TaN_x would segregate off from TiO_2 and form a separate phase.

The atomic percentages of Ta, Ti, O and N were calculated using the folowing equation:

$$\% X = 100 \times \left(\frac{S_X}{S_{Ta} + S_{Ti} + S_O + S_N}\right)$$

The S_x is the normalized peak area associated to each element. For normalization, the raw peak area was divided to the sensitivity factor. The sensitivity factore of Ti, Ta, N, and O are 52.013, 75.608, 12.231, and 18.643, respectively.

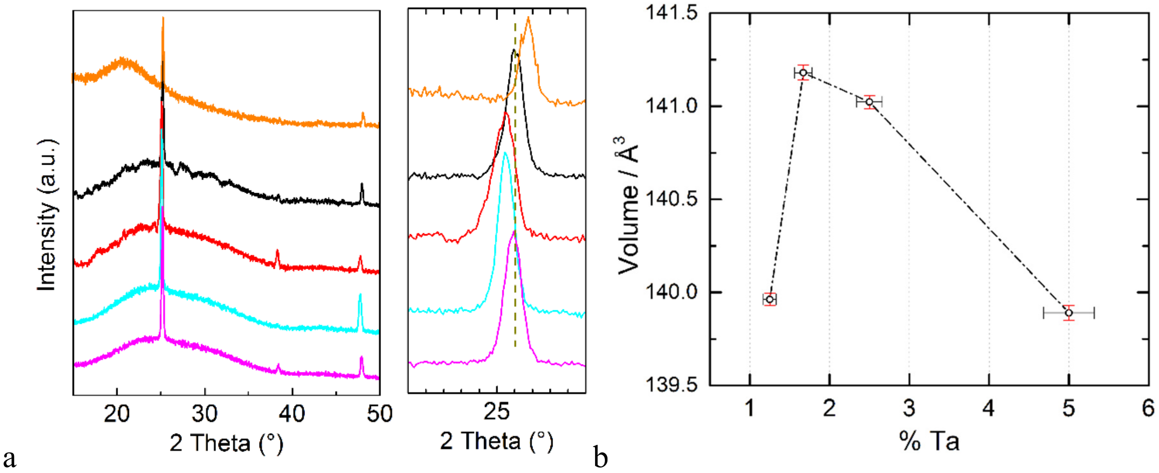

Figure S4. a) XRD and b) calculated cell volume of TiO_2 as a function of % Ta doping. Un-doped TiO_2 (orange). % 5.0 Ta ± 0.32 (pink), % 2.25 Ta ± 0.0.16 (Cyan), % 1.67 Ta ± 0.11 (red) and, % 1.25 Ta ± 0.08 (black).

The diffraction patterns were unambiguously matched to anatase TiO_2. Since the ionic radii of $Ta^{5+}$is slightly larger than Ti^{4+}(0.64 vs 0.60),[6] therefore, upon doping TiO_2 with Ta the unit cell would slightly expand. The films with low and high % Ta doping as well as un-doped samples

have almost the same peak positions. This observation suggests that these two films have the lowest Ta incorporation. The slight peak shift of lightly doped film, % 1.25 Ta doping, can be ascribed to the low concentration of Ta to effectively make a detectable alteration in anatase structure. On the other hand, the heavily doped film, % 5 Ta doping, has a high concentration of Ta which makes it energetically more favorable to form a separate phase of tantalum(oxy)nitride. This is consistent with the extra N and Ta peaks observed in XPS (Figure S17). On the other hand, For the films with % Ta doping in between, however, the diffraction peaks are shifted to the lower angles. GSAS[7,8] was used to calculate the cell parameters. The calculated volumes vs % Ta doping level are shown in Figure S20b.

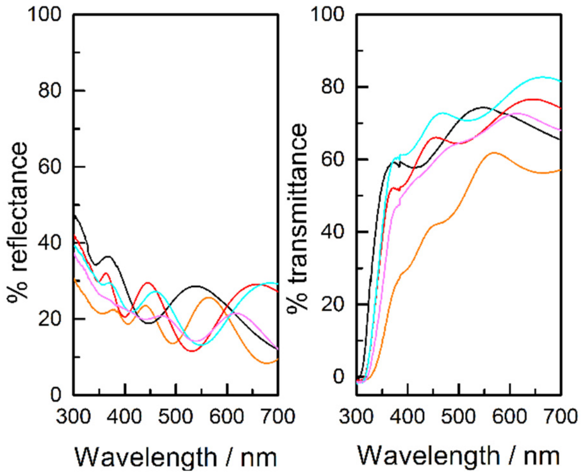

Figure S5. a) reflectance b) corrected transmittance of TTO as a function of concentration of Ta. Films were annealed in ammonia at 750 °C for 30 min. 200/1 (pink), 150/1 (Cyan), 100/1 (red), 50/1 (black), and un-doped TiO_2 (orange).

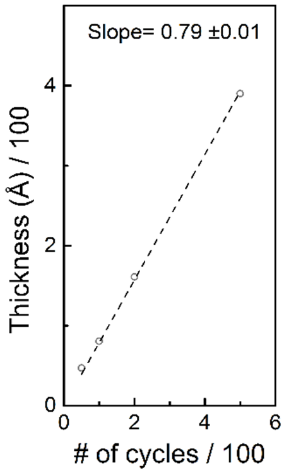

Figure S6. Thickness of TaO_x a *vs* the number of cycles deposited at 250 °C.

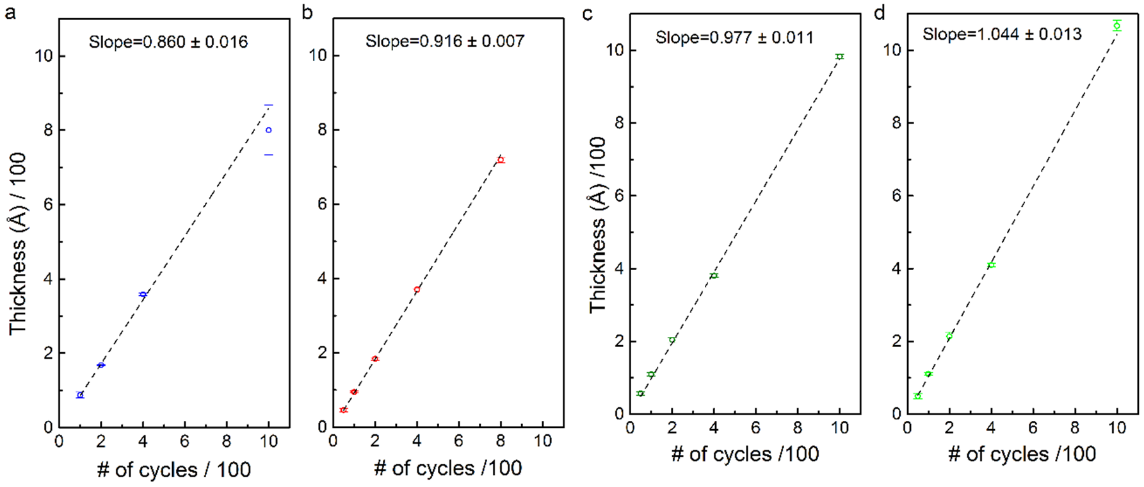

Figure S7. Thickness of TaO_xN_y *vs* number of cycles deposited at a) 175 °C, b) 200 °C, c) 250 °C, and d) 280 °C.

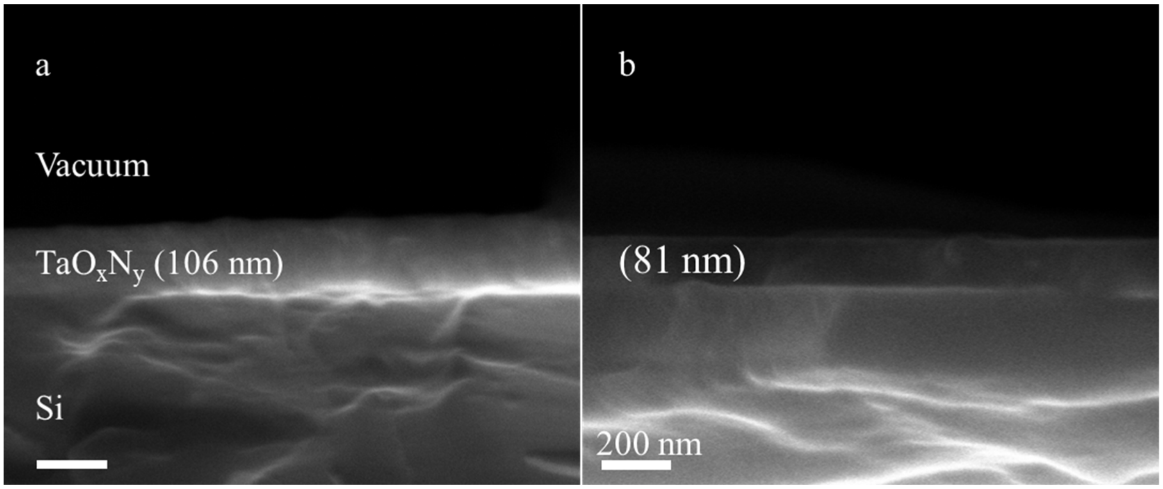

Figure S8. Cross section SEM image of 1000 cycles of TaO_xN_y deposited at a) 280°C, b) at 175 °C.

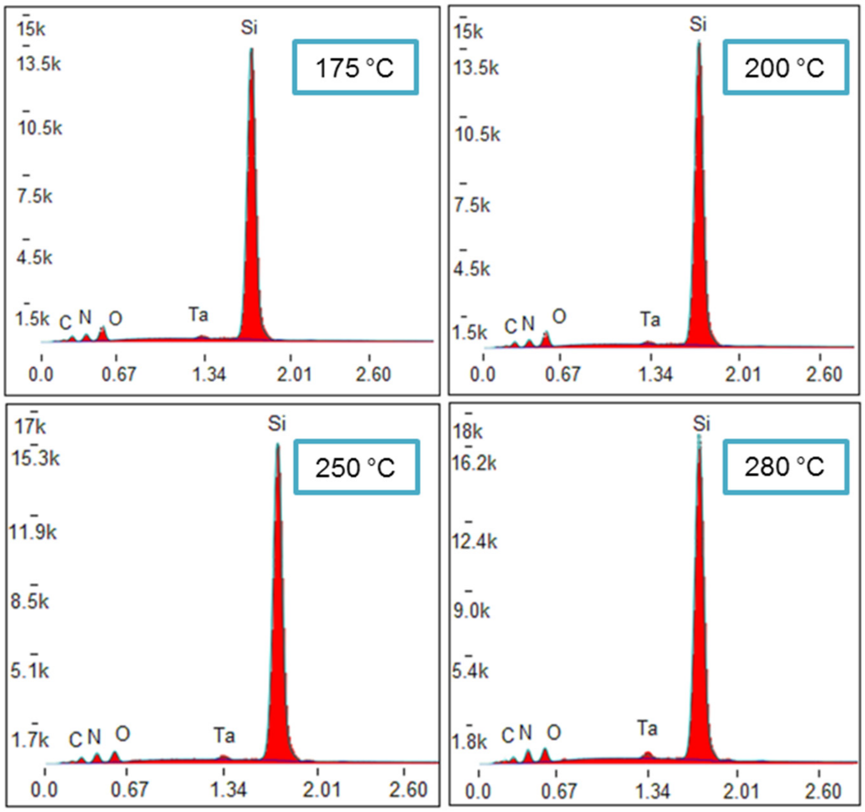

Figure S9. EDS spectrum of as-deposited TaO_xN_y as a function of deposition temperature. Silicon wafer with 16 Å native SiO_2 was used as the substrate. Ta main peak is buried under large Si signal.

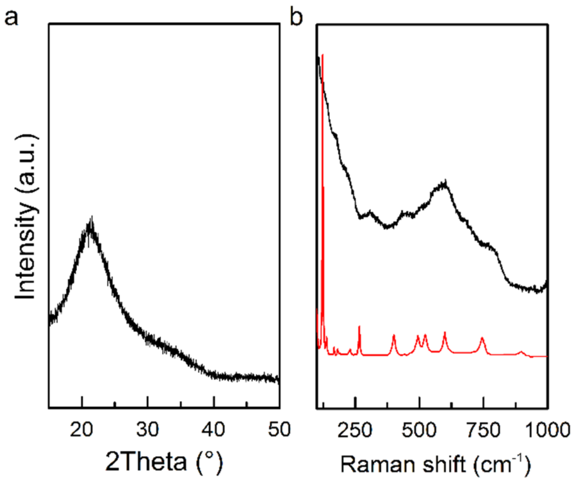

Figure S10. a) XRD and b) Raman scattering of 100 nm TaO_xN_y deposited on FTO at 250 °C.

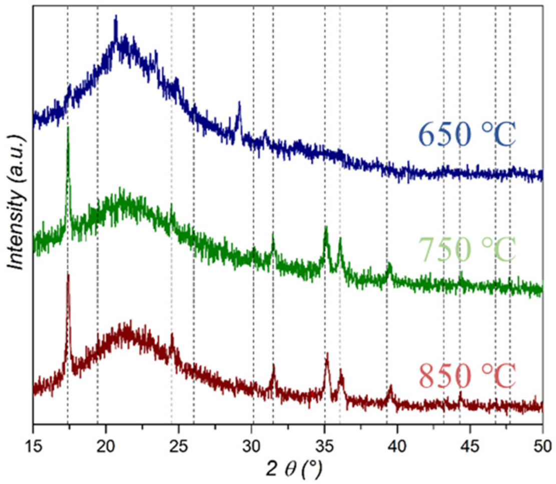

Figure S11. The XRD of TaO_xN_y, after annealing in ammonia at different temperature for 10 hours. The vertical dashed lines represent the brag position of Ta_3N_5 with PDF number of 01-089-5200.

To eliminate the effect of flow rate, high throughput ammonia (~ 500 SCCM) was used and the annealing temperature and duration were optimized. Based on the XRD, the minimum temperature required to form pure phase Ta_3N_5 was found to be 750 °C.

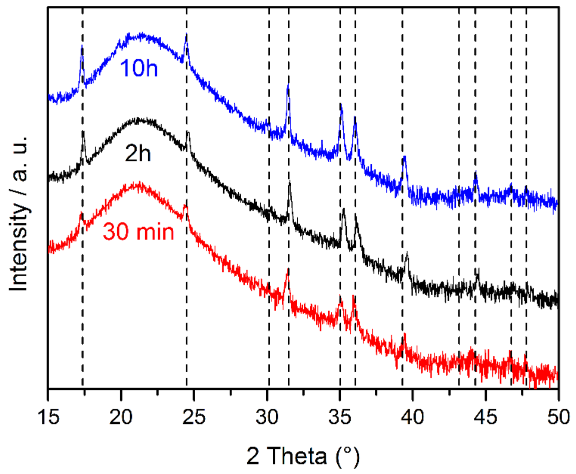

Fig. S12. a) The XRD pattern of TaO_xN_y deposited film after annealing at 750 °C for different durations. The vertical dashed lines represent brag position of Ta_3N_5 with PDF number of 01-089-5200.

Optimization of annealing duration was done by soaking temperature at 750 °C for different durations from 10 hours to 30 minutes. Evidently, the formation of pure Ta_3N_5 is completed after 30 minutes ammonolysis. However, increasing the annealing duration, results in a more well-defined and sharper peaks (more crystalline films). All the following films were annealed for 2 hours unless otherwise mentioned.

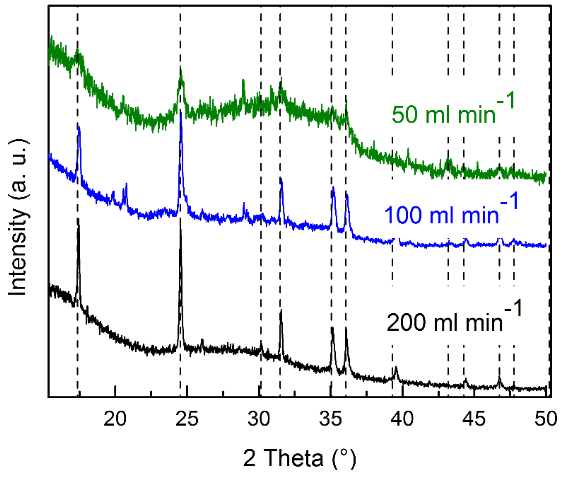

Figure S13. XRD of TaO_xN_y deposited film after ammonolysis at 750 °C for 2 hours as a function of ammonia flow rate. The vertical dotted lines represent the brag position of Ta_3N_5 with PDF number of 01-089-5200.

The effect of the ammonia flow rate on crystallinity and phase purity of the films were studied by annealing at 750 °C for 2 hours with different flow rate of ammonia. From Figure S9, films annealed at low flow rate of ammonia, e.g. 50 and 100 ml min^{-1}, had poor crystallinity and were comprised of impure phases (possibly TaON). On the other hand, for the flow rate more than 200 ml min^{-1}, the observed peaks were sharper (more crystalline) and Ta_3N_5 was the only detectable phase. This observation is consistent with previous study, where thin films of TaO_x and Ta_3N_5 were prepared on Ta foil. It was found that at lower flow rates the formation of TaON is more favorable where at higher flow rates only films are totally nitridized to Ta_3N_5. [2] Here we found that the optimum ammonolysis conditions to form pure and highly crystalline Ta_3N_5, is annealing at 750 °C for 2 hours with flow rate of ammonia of ≥ 200 ml.min^{-1}.

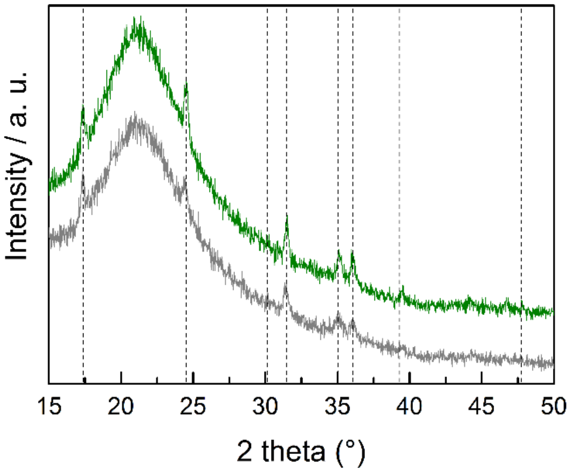

Figure S14. XRD of 50 nm ALD deposited TaO_xN_y (green) and TaO_x (gray) films after ammonolysis at 750 °C for 30 minutes as a function. The vertical dotted lines represent the brag position of Ta_3N_5 with PDF number of 01-089-5200.

The XRD of the ALD deposited TaO_x and TaO_xN_y are compared in Figure S10. As it can be seen the ALD deposited TaO_x were completely nitridized to Ta_3N_5 after ammonolysis at the same at 750 °C for 30 minutes.

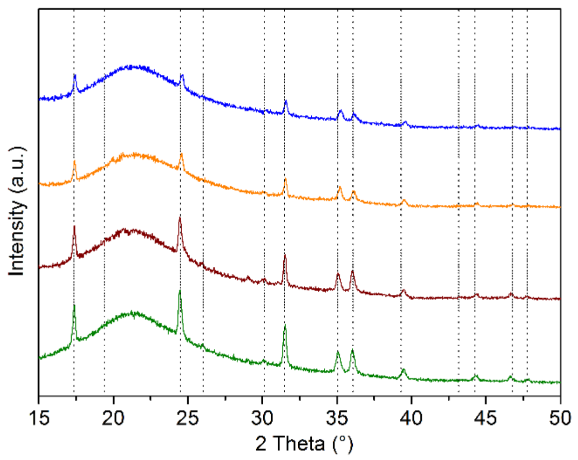

Figure S15. XRD of Ta_3N_5 with different thickness after ammonolysis. The film thicknesses are: 50 nm (blue), 70 nm (orange), 99 nm (maroon), and 122 nm (green). The vertical dashed lines represent Brag position for Ta_3N_5 with PDF number of 01-089-5200.

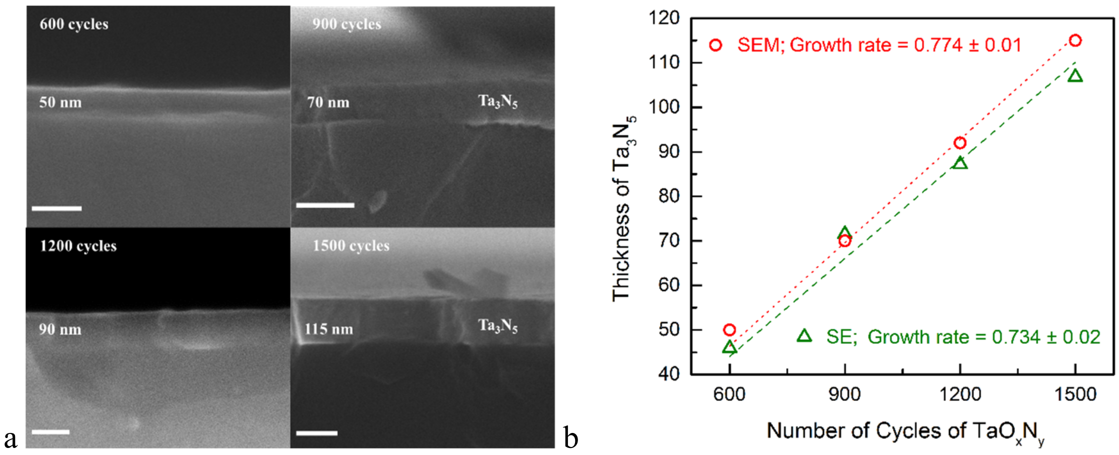

Figure S16. a) Cross section SEM image of Ta_3N_5 different number of cycles with the scale bar of 100 nm, b) Growth rate of pure Ta_3N_5 found by SE and cross section SEM.

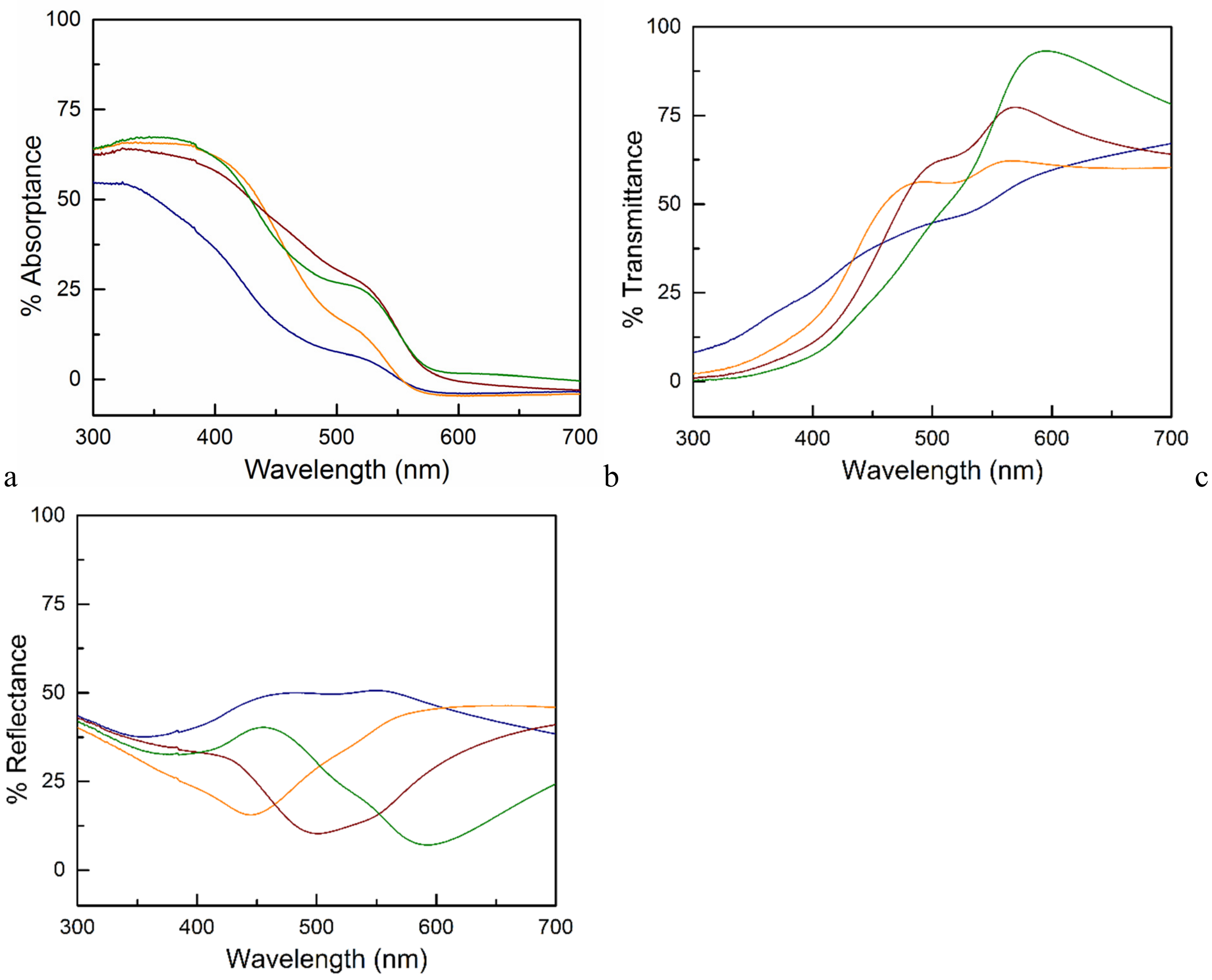

Figure S17. a) absorptance, b) % transmittance, and c) % reflectance of thin films of Ta_3N_5 as a function of thickness, 50 nm (blue), 70 nm (orange), 99 nm (red), and 122 nm (green).

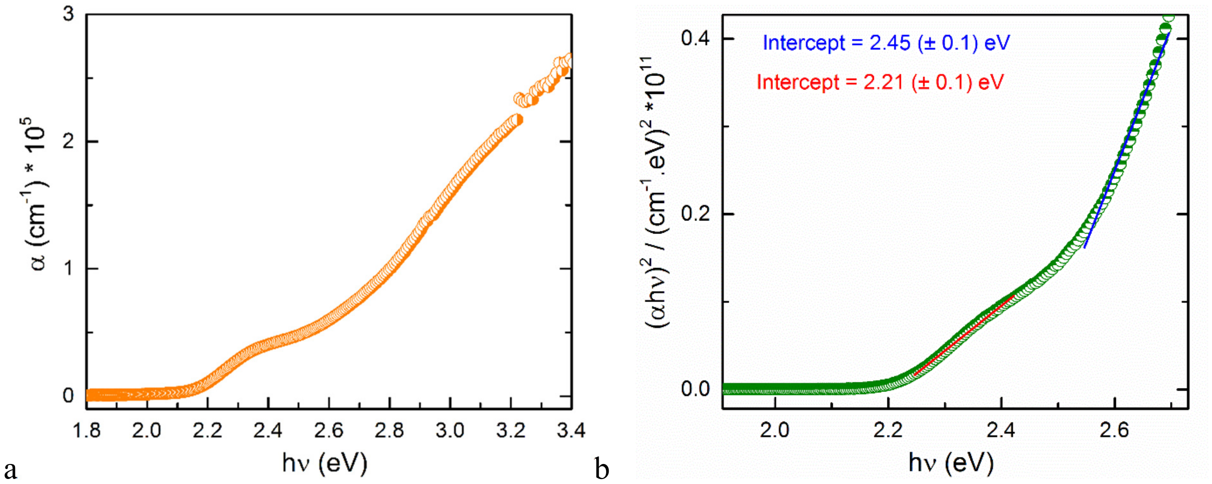

Figure S18. a) absorption coefficient of Ta_3N_5 as a function of wavelength, and b)Tauc plots for direct transitions.

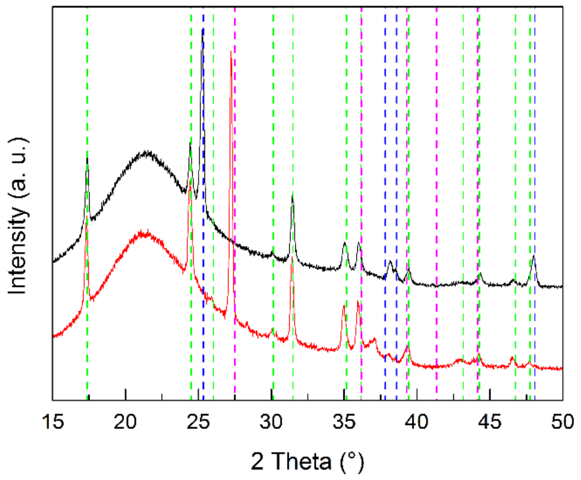

Figure S19. XRD of the films as a function of ammonolysis duration at 750 °C, 2 hours (red), and 30 minutes. The dashed line are the brag position of Ta_3N_5 (green), anatase TiO_2 (blue), rutile TiO_2 (pink).

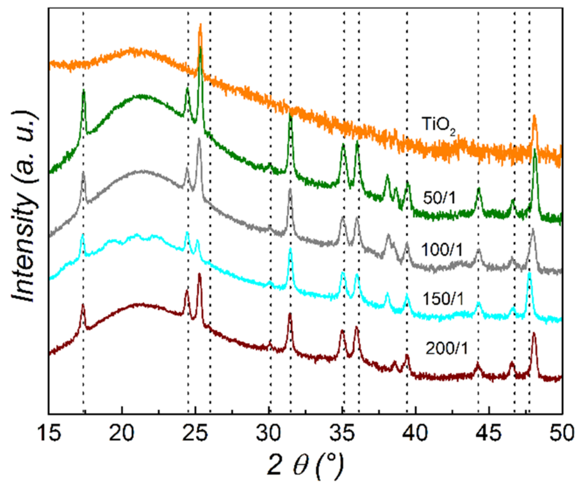

Figure S20. a) XRD of 40 nm of Ta_3N_5 on TTO with different Ta concentration after annealing in ammonia at 750 °C for 30 min. The vertical dashed lines represent the Bragg position of Ta_3N_5 with PDF number of 01-086-1155.

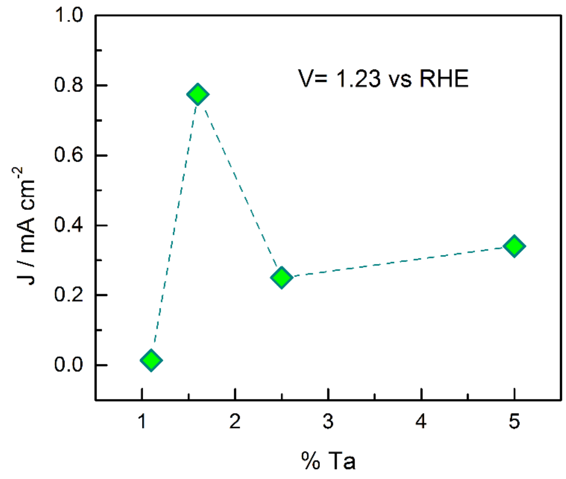

Figure S21. The photocurrent of 70 nm Ta_3N_5 on TTO at 1.23 V vs RHE as a function of Ta percentage in TTO.

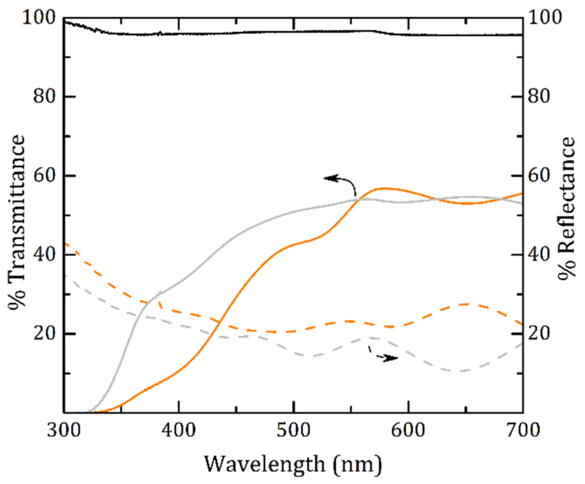

Figure S22. Transmittance and reflectance spectrum of electrodes with 5 % Ta-doped TiO_2 ~40 nm either TaOxNy ALD-deposited (Orange) or TaO_x ALD-deposited (gray) after ammonolysis at 750 °C for 30 minutes.

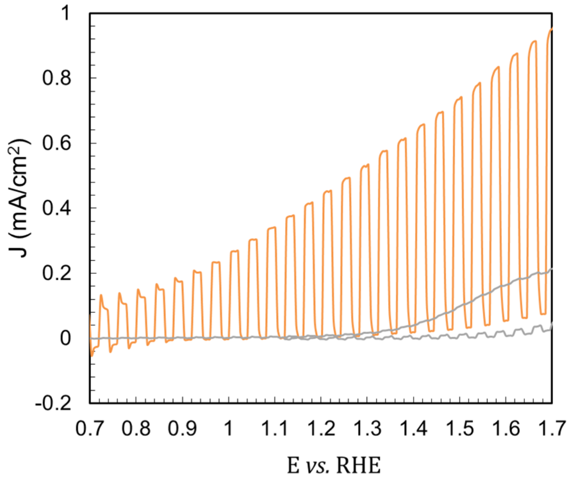

Figure S23. PEC performance of TaN_x (orange) or TaO_x (gray) on 5% Ta-doped TiO_2(100nm)/quartz after annealing in ammonia at 750 °C for 30 min level under 1 sun illumination and 0.5 M K_2HPO_4 phosphate solution with pH13. Note: prior to PEC water oxidation measurements, for all the electrodes, CoPi was photo-electrodeposited at 1.06 V vs RHE for 60s.

References

(1) Young, K. M. H.; Hamann, T. W. *Chem. Commun. (Camb).* **2014**, *50* (63), 8727–8730.

(2) Hara, M.; Chiba, E.; Ishikawa, A.; Takata, T.; Kondo, J. N.; Domen, K. *J. Phys. Chem. B* **2003**, *107* (48), 13441–13445.

(3) Yoon, J.-W.; Sasaki, T.; Koshizaki, N.; Noguchi, Y.; Miyayama, M. *Electrochem. Solid-State Lett.* **2004**, *7* (7), A172.

(4) Chen, T.-T.; Liu, H.-P.; Wei, Y.-J.; Chang, I.-C.; Yang, M.-H.; Lin, Y.-S.; Chan, K.-L.; Chiu, H.-T.; Lee, C.-Y. *Nanoscale* **2014**, *6* (10), 5106–5109.

(5) Yue-Ping, H.; Yan, H. *Chinese Phys. Lett.* **2009**, *26* (3), 036402.

(6) Shannon, R. D. *Acta Crystallogr. Sect. A* **1976**, *32* (5), 751–767.

(7) EXPGUI, a graphical user interface for GSAS http://www.ncnr.nist.gov/xtal/software/EXPGUI_reprint.pdf (accessed Aug 3, 2015).

(8) GENERAL STRUCTURE ANALYSIS SYSTEM http://www.ccp14.ac.uk/ccp/ccp14/ftp-mirror/gsas/public/gsas/manual/GSASManual.pdf (accessed Aug 3, 2015).
